# Host Age Prediction from Fecal Microbiota Composition in Male C57BL/6J Mice

**DOI:** 10.1128/spectrum.00735-22

**Published:** 2022-06-08

**Authors:** Adrian Low, Melissa Soh, Sou Miyake, Henning Seedorf

**Affiliations:** a Temasek Life Sciences Laboratory, Singapore, Singapore; b Department of Biological Sciences, National University of Singapore, Singapore, Singapore; Nanchang University

**Keywords:** aging, gut microbiota, mouse microbiota

## Abstract

The lifelong relationship between microorganisms and hosts has a profound impact on the overall health and physiology of the holobiont. Microbiome composition throughout the life span of a host remains largely understudied. Here, the fecal microbiota of conventionally raised C57BL/6J male mice was characterized throughout almost the entire adult life span, from “maturing” (9 weeks) until “very old” (112 weeks) age. Our results suggest that microbiota changes occur throughout life but are more pronounced in maturing to middle-age mice than in mice later in life. Phylum-level analysis indicates a shift of the *Bacteroidota*-to-*Firmicutes* ratio in favor of *Firmicutes* in old and very old mice. More *Firmicutes* amplicon sequence variants (ASVs) were transient with varying successional patterns than *Bacteroidota* ASVs, which varied primarily during maturation. Microbiota configurations from five defined life phases were used as training sets in a Bayesian model, which effectively enabled the prediction of host age. These results suggest that age-associated compositional differences may have considerable implications for the interpretation and comparability of animal model-based microbiome studies. The sensitivity of the age prediction to dietary perturbations was tested by applying this approach to two age-matched groups of C57BL/6J mice that were fed either a standard or western diet. The predicted age for the western diet-fed animals was on average 27 ± 11 (mean ± standard deviation) weeks older than that of standard diet-fed animals. This indicates that the fecal microbiota-based predicted age may be influenced not only by the host age and physiology but also potentially by other factors such as diet.

**IMPORTANCE** The gut microbiome of a host changes with age. Cross-sectional studies demonstrate that microbiota of different age groups are distinct but do not demonstrate the temporal change that a longitudinal study is able to show. Here, we performed a longitudinal study of adult mice for over 2 years. We identified life stages where compositional changes were more dynamic and showed temporal changes for the more abundant species. Using a Bayesian model, we could reliably predict the life stages of the mice. Application of the same training set to mice fed different dietary regimens revealed that life-stage age predictions were possible for mice fed the same diet but less so for mice fed different diets. This study sheds light on the temporal changes that occur within the gut microbiota of laboratory mice over their life span and may inform researchers on the appropriate mouse age for their research.

## INTRODUCTION

The gut microbiome is known to exert wide-ranging effects on host health (1). As such, understanding the dynamics of the murine gut microbiome is important for mouse model-based research. Age-related studies in humans and mice have shown that the gut microbiome differs at different phases of life (2, 3). While compositional variability can be attributed to factors such as housing and diet (4, 5), age-related factors such as host immunity-gut microbiota interactions are more likely to affect gut homeostasis and host health under controlled conditions (6). Over a host life span, other age-related changes include behavior (7), physiology (8), cellular biochemistry, and susceptibility to diseases (9, 10). In spite of this, the selection of younger mice for research is often based on practicality over host biology (11).

Longitudinal studies are rarely performed unlike cross-sectional studies as the latter are more feasible to conduct. Cross-sectional studies of the murine gut microbiome are generally focused on the early or later years of the murine life (2, 12, 13). One such study examined the gut microbiomes of “young” (24-week-old), “middle-age” (84-week-old), and “very old” (122-week-old) female C57BL/6J mice with observed major shifts in nine of the most abundant bacterial families and functional genes that could affect host health (2). These shifts, if reproducible, suggest that life stage-specific microbiome composition could serve as a biomarker of host age. Nevertheless, the reproducibility of these shifts remains uncertain due to the small sample size and high interindividual variability, which may result in diverging microbiomes among mice of different batches (2, 4). Already, such age-related characteristic microbiotas have been purported for humans from middle to late adulthood (14) and human host age has been predicted from fecal microbiota using a machine learning model, albeit with low accuracy (15). Here, we hypothesize that host age may be predictable from the fecal microbiota of mice kept under laboratory conditions.

This study aims to elucidate the temporal changes in the gut microbiome of conventionally raised and widely used adult C7BL/6J mice. The longitudinal analysis of the murine gut microbiome throughout its entire adult life span provides a highly resolved compositional profile, indicates life stage-specific microbiome compositions, and may allow for a more specific selection of mouse models for research questions relevant to the host age.

## RESULTS

### Microbiome composition changes throughout life.

**FIG 1:**
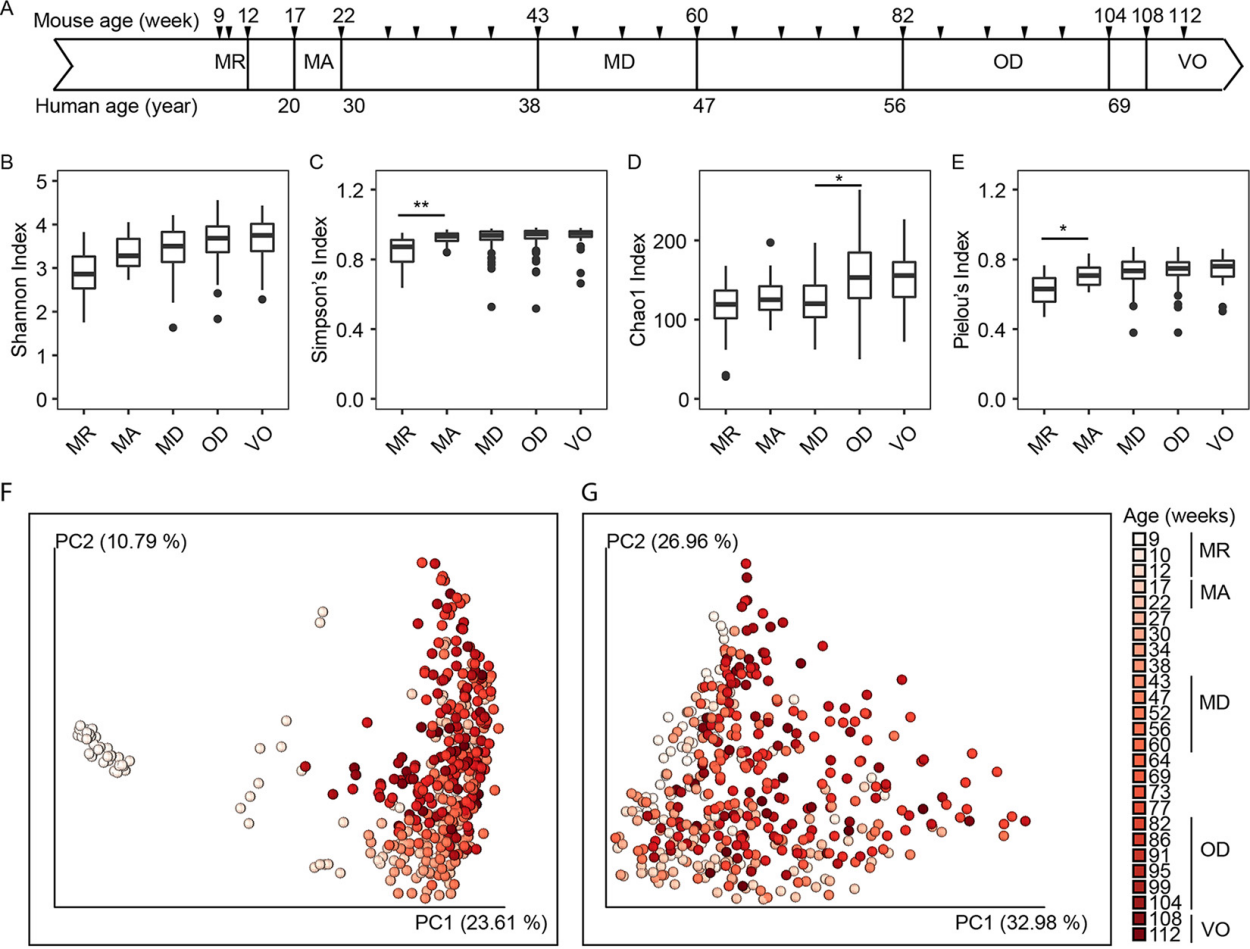
Longitudinal analyses of diversity and compositional changes to murine fecal microbiota over 103 weeks. (A) Outline of the longitudinal study of C57BL/6J male mice (*n *= 20) sampled at 4-week intervals from 9 to 112 weeks of age. Tick marks indicate sample time points (*n *= 26). (B to E) Alpha-diversity measures of fecal microbiota by murine life phases. Asterisks represent significant differences between adjacent life phases determined using the Wilcoxon signed-rank test: *, FDR-corrected *P* values of <0.05; **, FDR-corrected *P* values of <0.01. (F and G) Principal-coordinate analysis plots of Bray-Curtis dissimilarity (F) and weighted-UniFrac distances (G). The variance for each principal coordinate (PC) axis is shown in parentheses. MR, maturing; MA, mature; MD, middle age; OD, old; VO, very old.

The fecal microbiotas of 20 9-week-old C57BL/6J mice were characterized at regular intervals over 103 weeks (Fig. 1A shows the experimental timeline and defined life phases, and Fig. S1 in the supplemental material shows the survival curve). The differences in alpha-diversity between successive life phases were compared using Shannon, Simpson, Chao1, and Pielou’s evenness indices (Fig. 1B to E). There was an apparent increase in mean alpha-diversity for all four indices from “maturing” (MR) to “mature” (MA) mice, but the increase was significant only in Simpson’s diversity and Pielou’s evenness indices (see Tables S1A to E for Wilcoxon signed-rank tests). The alpha-diversity of fecal microbiota was largely stable from the MA to the “very old” (VO) phase, except for a significant increase in rare amplicon sequence variants (ASVs) (Chao1 richness) from “middle age” (MD) to “old” (OD) (Fig. 1D). Linear mixed-effects (LME) models were used to identify differences in alpha-diversity between mice in their first year (9 to 47 weeks old) and those in their second year (52 to 112 weeks old) (Fig. S2). Overall, there was an upward trend in alpha-diversity for all four indices as the mice aged (see Table S1F for LME results). Based on the slopes of the LME models, Shannon diversity increased in a similar trajectory in both years. Simpson’s diversity and Pielou’s evenness indices increased significantly more in the first year than in the second year. In contrast, Chao1 richness did not increase until the second year. Collectively, the indices indicate that species evenness was the primary change between the MR and MA phases and that species richness was accountable for the change between the MD and OD phases.

The temporal change in beta-diversity with respect to ASVs (Bray-Curtis) and phylogeny (weighted UniFrac) can be seen from the respective principal-coordinate analysis (PCoA) plots (Fig. 1F and G). Although the first principal coordinate, PC1, explained most of the variance for both matrices (rho_Bray-Curtis_ = 0.389, *P = *2.2 × 10^−16^; rho_weighted UniFrac_ = 0.371, *P = *1.5 × 10^−15^), PC3 showed the best Spearman correlation with age (rho_Bray-Curtis_ = 0.646, *P = *2.2 × 10^−16^; rho_weighted UniFrac_ = 0.486, *P = *2.2 × 10^−16^). Beta-diversity comparison of distances between successive time points was performed to detect intergroup longitudinal changes, and LME was used to test whether fixed effects of life phase on beta-diversity changed over time. The analysis revealed disparate rates of longitudinal change and disparities between the two beta-diversity measures. There was a significant decrease in the rate of Bray-Curtis dissimilarity from the MA phase until the first intermediate period where the microbiota was more stable (Fig. S3; see Table S2A for LME results). In contrast, the change in weighted-UniFrac distance in MA mice was more gradual. The microbiota fluctuated during the second intermediate period between the MD and OD phases for weighted-UniFrac distances but not for the Bray-Curtis index. At the OD phase, both beta-diversity measures have significantly different rates of change than the other life phases. Permutational multivariate analysis of variance (PERMANOVA) tests of Bray-Curtis dissimilarity and weighted-UniFrac distances between adjacent life phases showed that microbiotas from MR to OD mice were distinct while those from OD and VO mice did not differ significantly (see Table S2B for PERMANOVA test of Bray-Curtis dissimilarity between life phases and Table S2C for PERMANOVA test of weighted-UniFrac distance between life phases). Progression toward a more stable microbiota with age is observable longitudinally, as the number of significantly different pairs of microbiotas decreased with age (Fig. S3; see Table S2D for PERMANOVA test of Bray-Curtis dissimilarity between time points and Table S2E for *P* values of the weighted-UniFrac distance between time points). Taken together, the results indicate that the murine gut microbiota undergoes more compositional changes in the first year than the second year.

**FIG 2:**
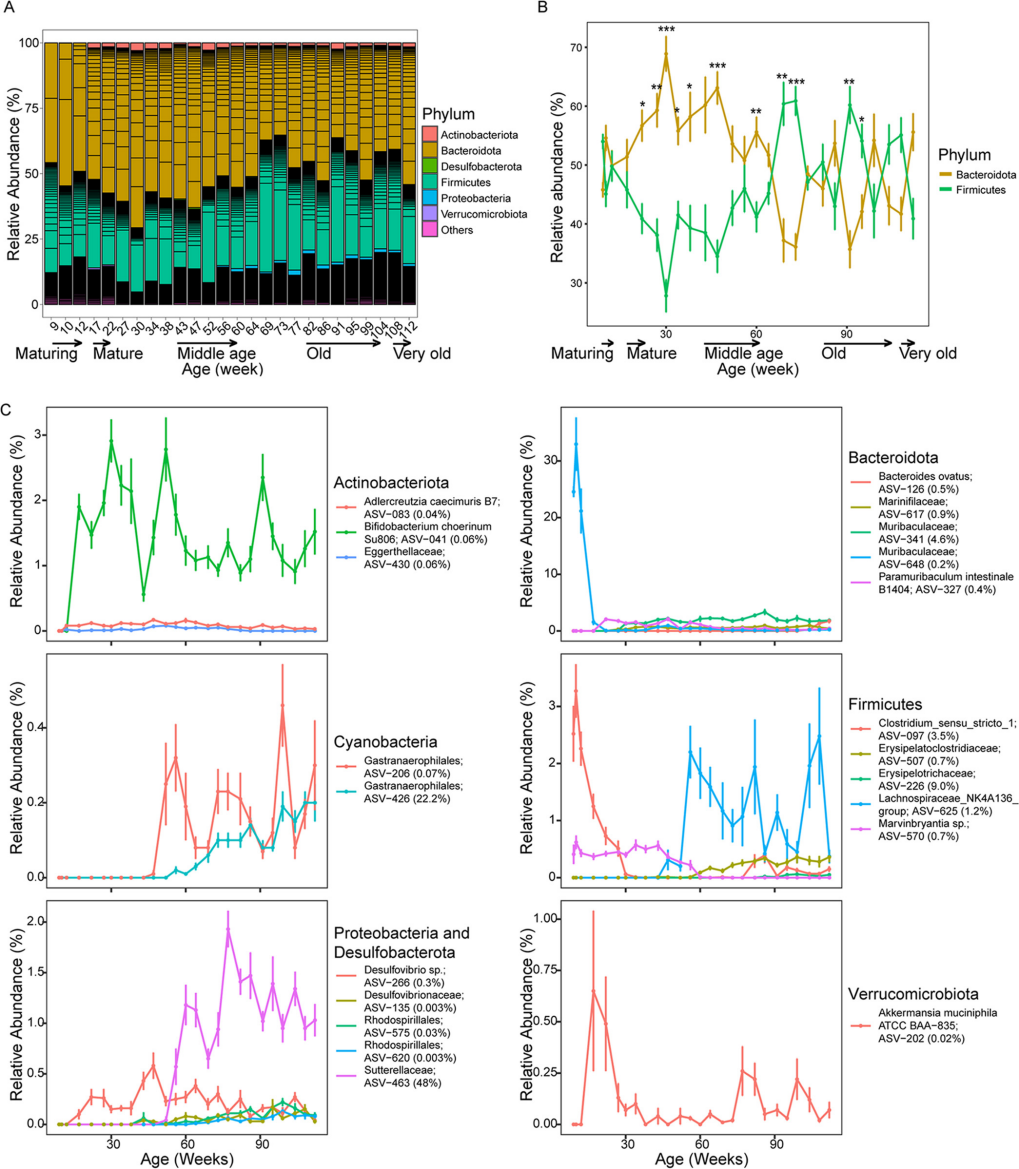
Compositional changes to the fecal microbiota of C57BL/6J male mice from 9 to 112 weeks of age. (A) Phylum groupings of ASVs with ≥0.5% relative abundance. *Cyanobacteria* and *Patescibacteria* have ASVs with <0.5% relative abundance and are grouped as “others.” Each ASV is denoted by a horizontal black line. (B) Changes in mean relative abundance (%) by the two major phyla. Asterisks represent significant differences between the two phyla for each time point determined using the Wilcoxon test: *, FDR-corrected *P* values of <0.05; **, FDR-corrected *P* values of <0.01; ***, FDR-corrected *P* values of <0.001. (C) Mean relative abundances of ASVs with top five importance scores (shown in parentheses) for each phylum based on a random forest regression model. *Actinobacteria*, *Cyanobacteria*, *Proteobacteria*, *Desulfobacterota*, and *Verrucomicrobiota* are represented by fewer than 5 predictive ASVs. Data points and error bars are the mean ± standard error of the mean, respectively. Relative abundances are based on an ASV table rarefied to 2,733 reads per sample (Table S3). Taxonomic assignments of >99% nucleotide identity for species level and 95% to 99% identity for genus level are based on top BLASTn hits, and those of <95% nucleotide identity for family level are based on the SILVA SSU database 138 release.

Differences in microbiome composition could be observed across phylogenetic levels. Figure 2A shows the relative abundance and SILVA 138-assigned phylum-level identity of 651 ASVs with ≥0.5% mean relative abundance at any time point over the 103-week study (see Table S3 for ASV table and taxonomic identities). *Firmicutes* and *Bacteroidota* were the predominant phyla, with similar relative abundances at the MR phase (Fig. 2B). *Bacteroidota* increased in relative abundance more than *Firmicutes* did, especially from the MA phase (22 weeks old) to the MD phase (64 weeks old) (Fig. 2B). After the MD phase, the *Firmicutes*/*Bacteroidota* ratio changed in favor of the *Firmicutes* and both phyla were relatively similar in abundance at the OD and VO phases (Fig. 2B; see Table S4 for the Wilcoxon signed-rank test of *Firmicutes* and *Bacteroidota* for the same time points). *Actinobacteria*, *Desulfobacterota*, *Proteobacteria*, and *Verrucomicrobiota* increased in relative abundance in MA mice, albeit at less than 3% (Fig. 2A). *Patescibacteria* and *Cyanobacteria* were detected at low relative abundances (<0.5%) throughout adult murine life (Fig. 2A). No archaeal phylum was detected throughout the study.

**FIG 3:**
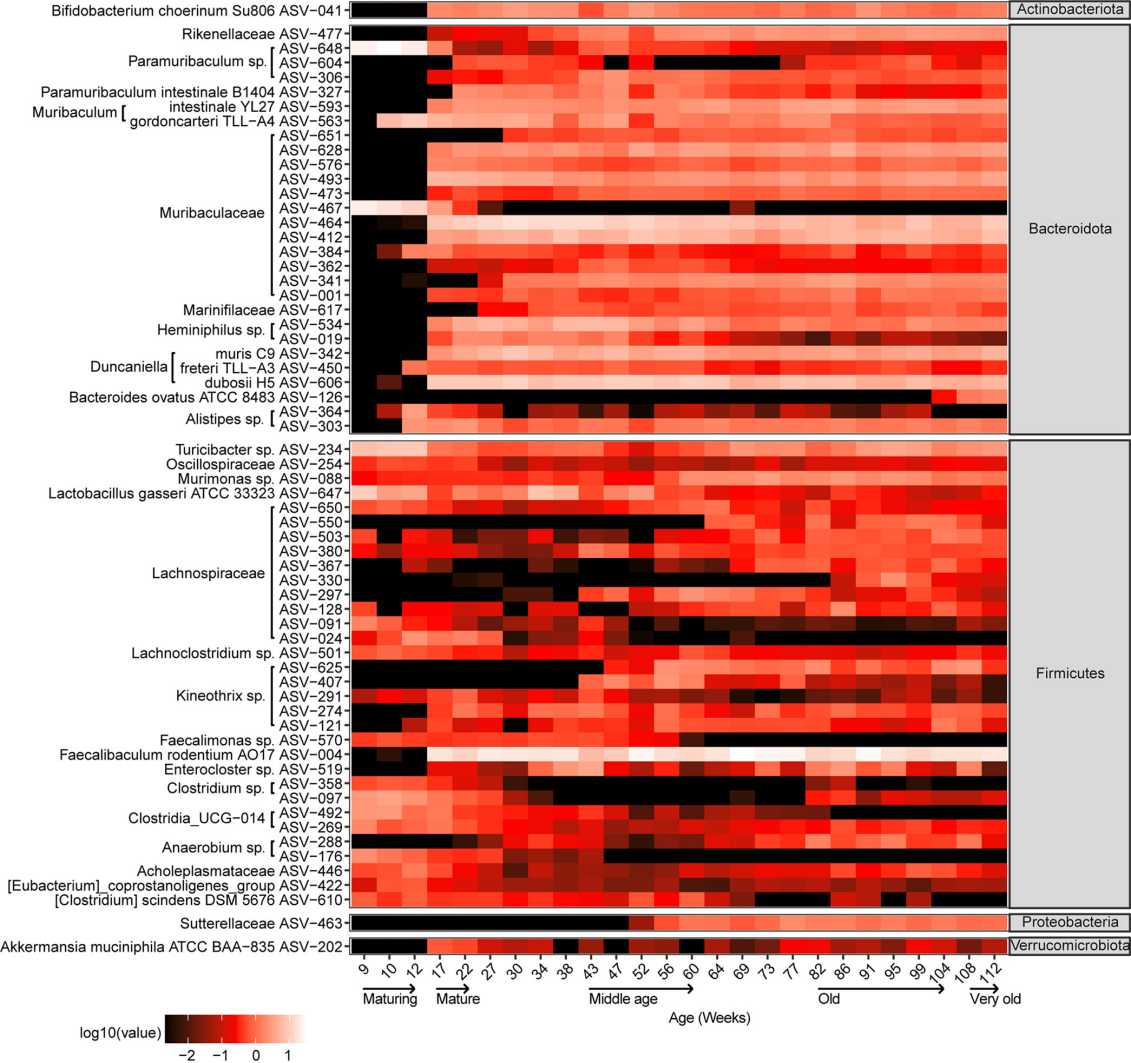
Heatmap of 63 ASVs across 26 time points from mice 9 to 112 weeks old. Each ASV has a mean relative abundance of ≥0.5% for one or more life phases. The total number of samples is 433 (see Table S5 for metadata). Taxonomic assignments with >99% nucleotide identity for species and 95% to 99% identity for genus level are based on top BLASTn hits, and those for <95% nucleotide identity for family level follow the annotation by the SILVA SSU database 138 release.

ASV-level analysis revealed 63 ASVs that constituted ≥0.5% mean relative abundance in one or more life phases (Fig. 3). Generally, ASVs for *Bacteroidota* and *Actinobacteria* were long-term commensals of the adult murine gut, becoming detectable after maturation until VO age. This was in contrast with the majority of *Firmicutes* ASVs, which were transient, with some detectable from 9 weeks but with different succession patterns, e.g., a few ASVs decreased in relative abundance as the mice aged (Fig. 3). *Proteobacteria*, represented by ASV-463 (*Sutterellaceae*), was in the MD phase (Fig. 2C). *Verrucomicrobiota*, represented by ASV-202 (Akkermansia muciniphila ATCC BAA-835), was most abundant during the MA phase (Fig. 2C and 3).

### Identification of ASVs predictive for host age.

The observation of the successional ASVs prompted the development of a random forest regression model to identify ASVs predictive of the 26 analyzed time points (16). This model shows a strong correlation (*r *= 0.983; *R*^2^ = 0.967; *P = *6.39 × 10^−65^; mean square error = 39.0) between predicted and actual microbiota for each time point. Of the 100 predictive ASVs, just six ASVs (ASV-463, ASV-426, ASV-226, ASV-341, ASV-097, and ASV-625) had a cumulative importance score of 89%, indicating that these ASVs have the greatest effect on the regression model (see Table S3 for importance score). The mean relative abundances of ASVs with the top five importance scores for each phylum are shown in Fig. 2C. Predictive *Firmicutes* ASVs varied in their successional patterns: ASV-097 (Clostridium_sensu_stricto_1) was more pronounced in earlier stages but diminished at 30 weeks of age, while ASV-570 (*Marvinbryantia* sp.) continued until 60 weeks of age. In contrast, ASV-507 (*Erysipelatoclostridiaceae*) and ASV-625 (*Lachnospiraceae_*NK4A136_group) were more prevalent from middle to late age than during earlier life phases. ASV-097 (Clostridium_sensu_stricto_1) was detectable only in mice 86 weeks and older (Fig. 2C). Of the *Bacteroidota*, ASV-648 (*Muribaculaceae*) was the most abundant during the MR phase but fell below detection in the MA phase. In contrast, three ASVs (ASV-327 [*Paramuribaculum intestinale*], ASV-341 [*Muribaculum* sp. J10; 96.0%], and ASV-617 [*Marinifilaceae* sp.]) became detectable from the MA phase and remained at a similar relative abundance throughout. ASV-126 (Bacteroides ovatus) was more abundant as the mice aged to VO than in mice in the earlier phases. Of the three predictive *Actinobacteria*, ASV-041 (Bifidobacterium choerinum Su806) and ASV-083 (Adlercreutzia caecimuris B7) were detectable throughout life phases, while ASV-430 (*Eggerthellaceae* sp.) was detectable at low relative abundance during the MD phase and in the later stages of murine life. Three of the *Proteobacteria* ASVs (ASV-575 and ASV-620, both *Rhodospirillales*, and ASV-463 [*Sutterellaceae*]) were middle to late successors. ASV-266 (*Desulfovibrio* sp.), belonging to *Desulfobacterota*, a phylum containing mostly sulfate reducers (17), was prevalent from the MA phase onwards, while ASV-135 (*Desulfovibrionaceae*) was more transient from the MD to VO phases (Fig. 2C). Two predictive *Cyanobacteria* ASVs belonging to *Gastranaerophilales* shared similar mid-succession patterns, reaching higher relative abundance only during MD and subsequently (Fig. 2C).

### Prediction of host age based on fecal microbiome composition.

**FIG 4:**
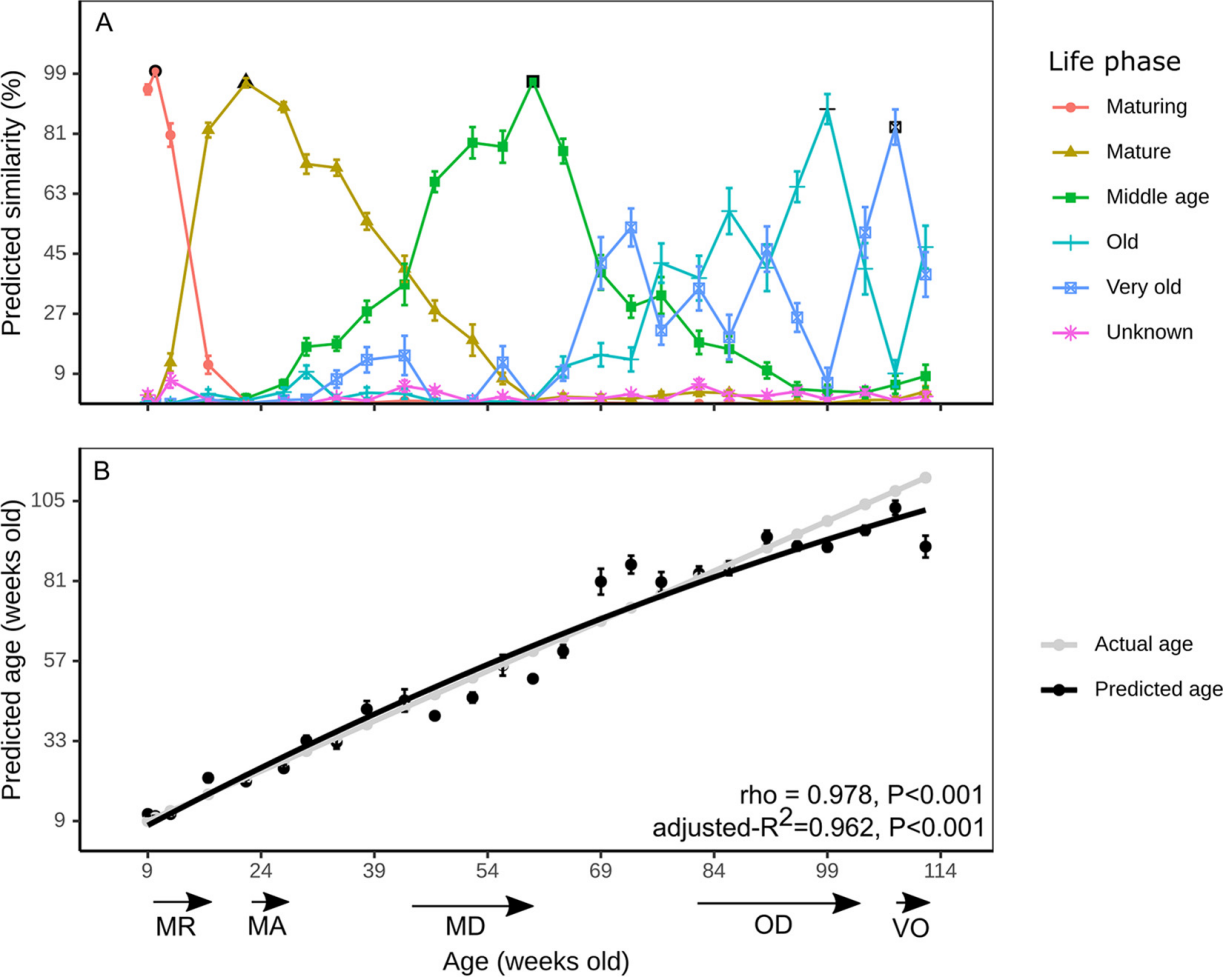
Host age estimation based on fecal microbiota composition. (A) SourceTracker v0.9.1-based prediction of probabilities for each time point for the five life phases. Symbols with a black outline show the time points used as the “source” for each life phase. (B) Correlation of predicted age to actual mouse age. The Spearman correlation coefficient (rho), adjusted *R*^2^ value of the polynomial regression, and respective *P* values are shown. Error bars are standard errors of the means and may be smaller than the symbol. MR, maturing; MA, mature; MD, middle age; OD, old; VO, very old.

The observed successional pattern during the aging process and the identification of predictive ASVs for specific time points prompted the question of whether the animal age can be inferred from a subset of fecal microbiota representative of the microbial community compositions in the five life phases. For this purpose, SourceTracker, which employs Bayesian statistics to determine the contribution of “source” communities to “sink” communities was applied to our longitudinal study (18). One time point from each of the five phases with the lowest number of significantly different microbiotas (false discovery rate [FDR]-corrected *P* values of <0.01; PERMANOVA test of Bray-Curtis dissimilarities) was selected as “source” communities for SourceTracker (Fig. S4). The regression model revealed that murine MR to MD life phases can in principle be predicted from fecal microbiotas but those between the OD and VO phases are less discernible (Fig. 4A). Correlating actual age with approximate predicted age revealed higher accuracy for younger mice (9 to 60 weeks old; Spearman ρ = 0.966, *P = *2.11 × 10^−8^) than for older mice (64 to 112 weeks old; Spearman ρ = 0.832, *P = *0.001) (Fig. 4B). This is congruent with the PERMANOVA analysis of beta-diversity matrices that showed the microbiome becoming more stable with aging mice (Fig. S4). It should be noted that SourceTracker has been found to underestimate the contribution of unknown sources to sinks under certain conditions (19), although this does not appear to affect the age/life phase prediction made by the model in the current study, as most time points are proportionally more similar to the source of the same life phase than to those of other life phases (Fig. 4A).

**FIG 5:**
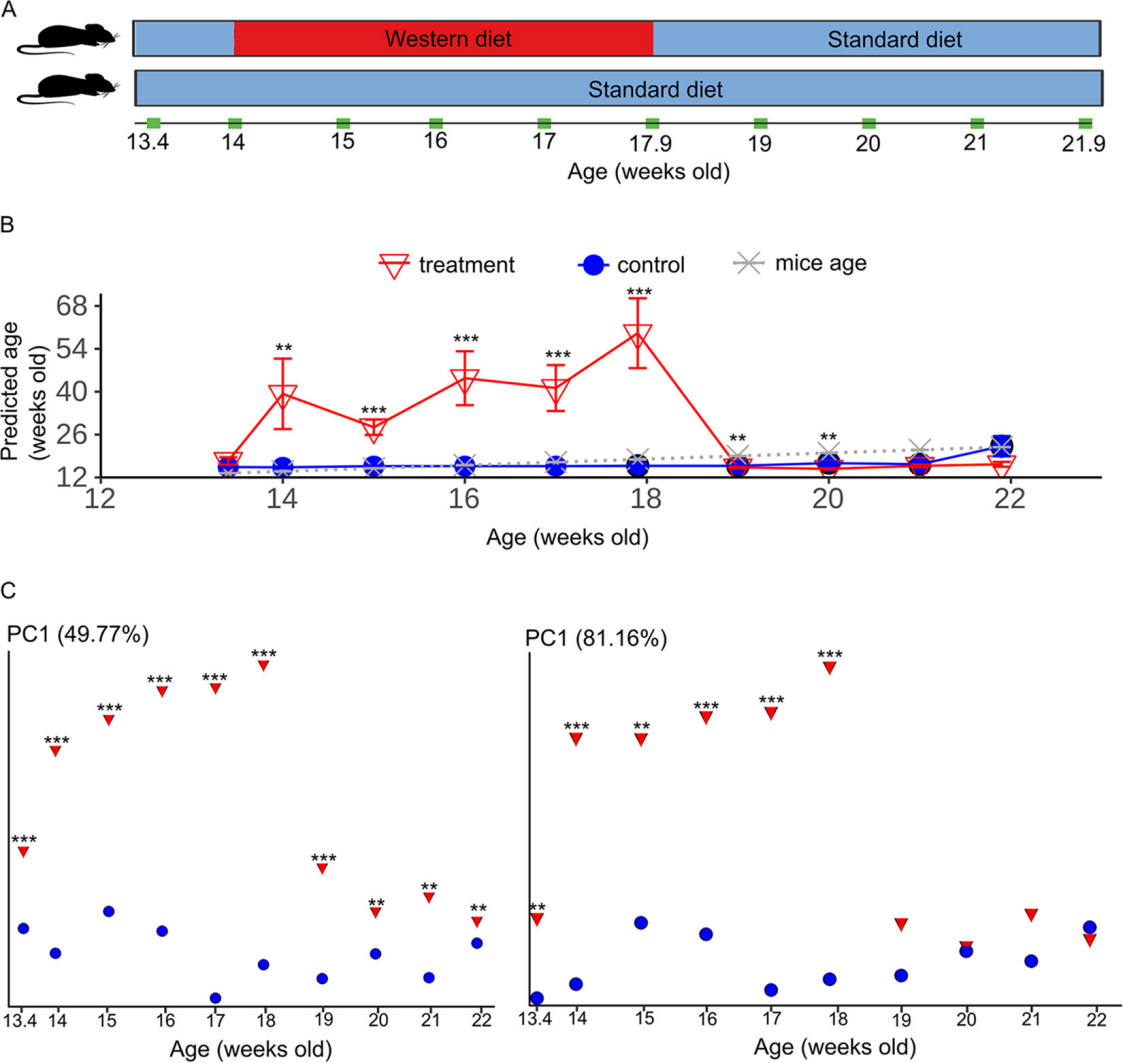
Age prediction and beta-diversity analyses of microbiota of a dietary study. (A) Schematic of the dietary experiment in which treatment mice 10 weeks of age (*n *= 12) were maintained on a standard diet until the start of a western diet at 14 weeks of age. Control group mice (*n *= 12) were fed only the standard diet. Treatment mice were fed standard chow at 18 weeks of age. Sampling time points are indicated in green. (B) Age prediction of mice. Black outlines indicate samples that were used as a “source” in addition to the training set used in the longitudinal study for SourceTracker v0.9.1. **, FDR-corrected *P* values of <0.01; ***, FDR-corrected *P* values of <0.001 based on the Wilcoxon ranked sum test. Error bars are standard errors of means. (C) Custom principal-coordinate analysis plots of Bray-Curtis (left) and weighted-UniFrac (right) distance matrices.

Host age prediction of a different batch of mice from a dietary treatment study was performed to examine the robustness of the training set (same five life phases) derived from the longitudinal study and a calibrated training set comprising the longitudinal study training set and control mice 17.9 to 21.9 weeks old (five time points) from the dietary treatment study. Treatment mice (*n *= 12) aged 14 weeks old were fed a western diet for 4 weeks and switched to a standard diet for another 4 weeks, while the control group was fed a standard diet throughout the experiment (Fig. 5A). Control mice 13.4 to 17 weeks old (mean ± standard deviation [SD] = 15.06 ± 1.3 weeks old) were predicted to be much older when the uncalibrated training set (26.9 ± 9.3 weeks old) was used than when the calibrated training set (17.8 ± 19.8 weeks old) was used (Fig. S5A). There were also more predicted 'sink' samples because of the calibrated training set than the uncalibrated training set (Fig. S5B). The validation justified the use of the calibrated training set to predict the ages of western diet-fed mice to adjust for batch variation and to study the dietary effects on host age prediction. The age prediction of mice fed a western diet was much higher (mean ± SD, 42 ± 11 weeks old) than that of mice fed a standard diet (mean ± SD, 16 ± 0.2 weeks old) during the period the mice were fed the western diet (5 time points) (Fig. 5B). After reverting to a standard diet, treatment mice showed predicted ages approaching chronological host age and control mouse age (Fig. 5B). This is congruent with PCoA plots of Bray-Curtis and weighted-UniFrac matrices, which showed the microbiotas converging after diet switch (Fig. 5C).

## DISCUSSION

It has been well established that the microbiomes of humans and animals change significantly in early life phases, e.g., after the weaning transition from milk to solid foods (12, 20, 21). Studies using gnotobiotic mice that received the murine gut microbiota of different ages have displayed undesirable and beneficial phenotypes, which highlights the need to understand the longitudinal changes in the gut microbiome of conventionally raised mice (12, 22, 23). In this study, we characterized the gut microbiome composition of conventionally raised adult male C57BL/6J mice at monthly intervals for 2 years, which corresponds to the approximate life span of this inbred strain (10). We observed microbiome variations during murine gut development not reported in previous cross-sectional or shorter longitudinal murine studies (2, 24, 25), such as a significant increase in species richness and/or evenness during early and late stages of life where shifts in relative abundance between phyla were observed (Fig. 2A). Interestingly, a similar increase in alpha-diversity of the swine gut microbiome was also observed during the growing years (26, 27), indicating that it may be an evolutionary trait that is conserved across species. The presented findings have potentially implications for research using mouse models in which the vast majority of mice used for experiments are aged between 8 and 12 weeks old (11). The temporal changes observed here will likely differ in female C57BL/6J mice owing to physiological differences such as hormones, which can affect gut microbiota diversity (28). Given the current study, mouse age should be selected carefully based on the research question.

Another notable observation is that the rate of change did not appear to be constant. There was a higher frequency of compositional change in the gut microbiome in the first year up to the MD phase (~47 weeks old), while the second year was marked by a reduced frequency of compositional change, especially at the lower taxonomic levels (i.e., fewer ASVs in succession and more ASVs in decline) from the OD to the VO phase. At this stage, the host may experience a decreased rate of glucose and fatty acid metabolism and reduced energy expenditure (29). Feed intake (normalized to body weight) has been shown to not differ significantly in mice from mature to very old age (30), which indicates that the feed intake has only a limited effect as an underlying cause for the observed changes. This could explain the absence of a significant difference in alpha-diversity between OD and VO mice (Fig. 1B). While it is generally agreed that a loss of diversity at old age may result in undesirable phenotypes such as inflammation or frailty in mice (2, 22) and increased frailty and reduced cognitive performance in humans (31, 32), our study suggests that a loss of gut diversity may not necessarily be associated with chronological age under controlled conditions. A meta-study of microbiota-based age prediction of humans (*n *= 8,959; 9 to 90 years old) showed a similar asymptotic regression curve for elderly individuals (>60 years old or OD phase mice) (Fig. 1A), supporting the hypothesis that prediction of life phase of a host, if not the chronological age, is possible from gut microbiota (controlled setting) or skin microbiota (uncontrolled setting) (15).

Our analyses have revealed similar (e.g., *Actinobacteria* and *Bacteroidota*) and variable (e.g., among *Firmicutes* ASVs) succession patterns, including long-term ASVs among the different phylogenetic lineages (Fig. 2 and 3). Notably, the beneficial Faecalibacterium rodentium (ASV-004) that emerged in mature mice is the predominant *Firmicutes* species (mean ± SD relative abundance, 17.5% ± 6.9% from 17 to 112 weeks old) over their life span (Fig. 3) (33). *Firmicutes* shared similar relative abundances with *Bacteroidota* at the OD and VO phases. However, prior to the late-stage shift in relative abundance, *Bacteroidota* relative abundance was significantly higher than that of *Firmicutes* over several time points from mature mice (Fig. 2B). This observation in mice is similar to previous studies of elderly humans that generally reported an age-related decrease in the *Firmicutes*-to-*Bacteroidota* ratio (34, 35). It is currently not possible to determine if these divergent patterns are specific to the host species, as similar observations have been shown for other factors, such as immune maturation or microbe-host interaction (36, 37), or whether it may potentially be an artifact, e.g., microbiota variation resulting from cross-sectional analyses. Comparisons of our data to other murine studies may be hampered since compositional differences may be influenced by many factors, including housing facilities (4).

The random forest regression model identified key ASVs, including some low-abundance ASVs, that are predictive of host age (Fig. 2C). Specifically, ASV-463 (*Sutterellaceae* of *Proteobacteria*) was most predictive for the temporal changes with succession at the MD stage of mouse life (Fig. 2C). *Parasutterella*, a genus of the *Sutterellaceae* family, is ubiquitous in the gut microbiomes of mammalian and human hosts and may benefit the host with bile acid maintenance and cholesterol metabolism (38). The successional pattern observed for most *Proteobacteria* ASVs, i.e., middle to late successors, is consistent with other murine and human gut microbiome studies (2, 24, 34, 39). *Desulfovibrio* sp. (ASV-266), a sulfate-reducing bacterium of *Desulfobacterota* previously classified as *Proteobacteria* (17), was detected from the MA to VO stages (Fig. 2C), supposedly performing an important role along with acetogens in lowering hydrogen partial pressure in the gut (40, 41). Notably, we did not detect methanogens, which could also contribute to hydrogen consumption in the gut, at any of the life phases. However, this is in line with previous studies that did not detect methanogen taxa in feces or along the gastrointestinal tract of adult laboratory mice. It would be interesting to see if the successional changes observed at the species level are conserved in other murine strains.

Our study clearly demonstrates the continuous changes of the gut microbiome and the different ASV succession patterns. The gut microbiomes of C57BL/6J mice largely followed host physiological development, but the periods of progression varied slightly (10). For example, gut microbiota took about 21 weeks before the transition from the MA to the MD phase, much longer than the transition from the MD to the OD phase (5 weeks). The reliable host age prediction showed that there was sufficient distinction even with a single “source” microbiota from each phase to delineate most fecal microbiomes from the MR to the OD phase. Limits to the prediction were observed for mice between the OD and the VO stage, where the microbiotas were too similar to be differentiated. The natural life span of mice sets limits to extend these experiments and to observe more small-scale dissimilarities between microbiomes of the very late stages. It also needs to be noted that these experiments were conducted in a well-controlled laboratory environment and the same standard laboratory feed was used for the entire duration. Using a different diet, alternating between diets, or performing other experimental modifications may also have lasting effects on the microbiome composition and its association with age, as previously reported (24). Our age prediction with fecal microbiota from a different batch of C57BL/6J mice with a subgroup fed a western diet showed that in the same strain of mice, at least, we could use a subset of control group mice to calibrate for batch variations in microbiota for SourceTracker. We could reliably estimate the ages of mice fed the same type of standard chow. Hence, the broad use of the longitudinal data set as a source is limited to mice with similar fecal microbiotas. Although compositional changes to the same diet-altered fecal microbiota have been reported (42), we showed that a western diet-altered fecal microbiota was predicted to be associated with much older mice than standard diet-fed mice (Fig. 5B). Interestingly, this observation is not entirely unexpected. A Western diet has been shown to increase the *Firmicutes*-to-*Bacteroidota* ratio (FBR) in humans and mice similar to the gradual increase in FBR associated with host age shown in the longitudinal study (43). Whether the underlying gut microbiota configuration could also contribute directly to the aging process remains to be investigated, as well as the use of other experimental diets. However, the diet-induced discrepancy between predicted age and chronological age could be of interest for the development of biomarkers or even therapeutics for healthy aging. The western diet-fed animals also highlight that accurate age prediction is not possible for microbiotas dissimilar to our training set. Hence, a new training set would be required for the myriad of factors such as housing facilities and murine strain that influence gut microbiota heterogeneity (4, 44).

In summary, this study shows that the fecal microbiome of laboratory mice changes substantially throughout the adult age. Consequentially, this has implications for the design of experiments where the microbiome can be considered a contributing factor affecting host physiology. Furthermore, this study highlights that the microbiome can serve as a biomarker of aging and that host age can potentially be inferred from microbiome composition.

## MATERIALS AND METHODS

### Animal husbandry, fecal sampling, and DNA extraction.

Experiments involving mice were approved by the Institutional Animal Care and Use Committee (IACUC number TLL-17-018) in accordance with National Advisory Committee for Laboratory Animal Research guidelines and were performed at Temasek Life Sciences Laboratory, Singapore, with supervision by trained veterinarians. Male C57BL/6J mice were purchased from InVivos (Singapore) at 63 days of age. Mice (*n = *20) for longitudinal analysis were kept in four cages of five mice each and maintained on standard chow (carbohydrate, 62.3%; protein, 25.5%; fat, 13.1%; PicoLab Rodent Diet 20; LabDiet, St. Louis, MO, USA) *ad libitum*. Nine-week-old mice (*n *= 24) from the dietary treatment study were sourced from the same vendor and fed the same standard chow until the experiment commenced (42). Fecal materials were sampled from all mice at 10 weeks of age and 12 weeks of age, followed by monthly 4- to 5-week intervals from 17 to 112 weeks of age except upon arrival (at 9 weeks of age), at which time a subsample of mice (*n *= 4) were sampled (Fig. 1). Fecal matter was collected directly from the anus using 2-mL sterile screw-cap tubes and flash-frozen in liquid nitrogen before storage at −80°C until DNA extraction. A bead-beating phenol chloroform DNA extraction method was used on all fecal samples as previously described (45).

### Amplicon sequencing of 16S rRNA genes.

A dual indexed 16S rRNA gene amplicon library was generated using primers 515F (46) and 806R (47) in triplicate PCRs per sample according to the protocol and indexes described for the Earth Microbiome Project (48). Illumina MiSeq sequencing was performed at the Genome Institute of Singapore according to the MiSeq reagent kit v2 (2 × 250 bp) preparation guide (Illumina, San Diego, CA, USA).

### Sequence processing and microbiota analysis.

The MiSeq fastq files were processed using QIIME 2 v.2021.4 (accessed on 1 November 2021) using “qiime tools import” (49). Default options were used for all QIIME 2 scripts unless stated otherwise. Forward primer sequences were removed from demultiplexed fastq files using the “qiime cutadapt trim-single” command. Forward reads for longitudinal and dietary studies were denoised together using the “qiime dada2 denoise-single” command for DADA2 (50) with “–p-trunc-len 176” as an option to truncate reads to 176 bp. ASVs in fewer than five samples were removed from analysis using the “qiime feature-table filter-features” command to minimize spurious reads. The command “qiime diversity core-metrics-phylogenetic” was used to rarefy the longitudinal and dietary studies to 2,733 and 9,778 reads per sample, respectively. The same command generated outputs for beta-diversity measures, including Bray-Curtis dissimilarity and weighted-UniFrac distance matrices and PCoA plots. Custom PCoA plots were generated using the “qiime emperor plot” command to plot samples grouped by age on the *x* axis against the first principal coordinate (PC1) on the *y* axis (51). Read counts for the dietary experiment were grouped by age using the “mean” option of the “qiime feature-table group” command. Alpha-diversity, and relative abundance and heatmap plots were generated using the phyloseq (52), ggplot2 (53), reshape (54), microbiome (55), genefilter (56), data.table (57), and patchwork (58) packages for R (59). Taxonomic identities for ASVs were assigned using the “qiime feature-classifier classify-sklearn” command against a trained classifier SILVA SSU for V4 region v.138 nonredundant 99% identity database (60). Bray-Curtis dissimilarity and weighted-UniFrac distance between successive time points were calculated using the “qiime longitudinal first-distances” command via the q2 longitudinal plugin (61). To identify ASVs that are predictive of the temporal changes, the “qiime longitudinal feature-volatility” command was used with “–p-n-estimators 100” and “–p-random-state 10” options that adopt the random forest regressor as a machine learning method (16, 61). To obtain updated taxonomic identities, selected predictive ASVs were annotated to the GenBank nonredundant database (accessed 28 November 2021) using the megablast function of BLASTn v.2.10.0+ (62, 63). SourceTracker v.0.9.1 was used to estimate mouse ages from an unrarefied ASV table with -b 100 (burnins), -n 10 (random restart), and -r (rarefied to 2,733 reads per sample) (18). As SourceTracker estimates the probability of a “sink” microbiota compared to a “source” microbiota, samples picked as a “source” were based on the time points with the fewest number of significantly different pairs, i.e., a more-similar microbiome for most samples, by using PERMANOVA analysis of the Bray-Curtis matrix. One representative time point was picked as a “source” for each of the five life phases. Samples selected as “sources” were also analyzed as “sinks.” An approximate age prediction for individual time points was performed by using the sum of the products of predicted life stage fractions and midpoints at each life phase:

$$y_{\text{age}}=\;a_{\text{MR}}x_{\text{MR}}+\;a_{\text{MA}}x_{\text{MA}}+\;a_{\text{MD}}x_{\text{MD}}+\;a_{\text{OD}}x_{\text{OD}}+\;a_{\text{VO}}x_{\text{VO}}$$

where *y*_age_ is the predicted age of the mouse based on fecal microbiome composition, *a* is the midpoint of the life stage in weeks old (i.e., MR = 10.5, MA = 19.5, MD = 51.5, OD = 93, and VO = 110 weeks old), and *x* is the predicted proportion of the life stage. For predicting mouse age of the dietary experiment, control mice from 17.9 to 21.9 weeks old were included in the longitudinal study training set to calibrate for batch microbiota variation. Samples with more than >30% uncertainty were removed from the age prediction analysis.

### Statistical analysis.

Wilcoxon signed-rank tests with Benjamini-Hochberg correction for alpha-diversity between life phases were performed using the “qiime longitudinal pairwise-differences” command and plotted using phyloseq (52), tidyr (64), and dplyr (65) packages for R (59). The Wilcoxon ranked sum test for between-treatment groups in the dietary study was performed using R (59). LME models were predicted using the “qiime longitudinal linear-mixed-effects” command (61). LME was used to test the effects of year (year 1 and year 2 time points) on alpha-diversity over time (age) as fixed effects. LME was used to test the effects of life phases on beta-diversity over time (age) as fixed effects. The same random intercept (mouse identity) and slope (age) were used as random effects for all LME models. Pairwise PERMANOVA tests were performed using the “qiime diversity beta-group-significance” QIIME 2 command based on 9,999 permutations with *P* values corrected using the Benjamini-Hochberg FDR method (66). Spearman correlations and polynomial regression for SourceTracker prediction between actual and predicted ages were performed using R (59). A *P* value of <0.05 is considered statistically significant for all tests except PERMANOVA, where a more stringent *P* value of <0.01 was used.

### Data availability.

MiSeq fastq files have been deposited in NCBI under BioProject number PRJNA503299 for the longitudinal study (see Table S5 in the supplemental material for BioSample accession numbers). BioSample accession numbers for a subset of samples from the dietary treatment study (BioProject number PRJNA503296) are specified in Table S6. The QIIME 2 codes/metadata/output files and SourceTracker input files are available in the GitHub repository at https://github.com/alow711/Seedorf_lab-Host-age-prediction.git.
