# Supplementary material for: Host Age Prediction from Fecal Microbiota Composition in Male C57BL/6J Mice
Source: Microbiol Spectr. 2022 Jun 8;10(3):e00735-22. doi: 10.1128/spectrum.00735-22 (PMC9241839; doi:10.1128/spectrum.00735-22)
Supplement: Supplemental file 1 — Supplemental material. Download spectrum.00735-22-s0001.pdf, PDF file, 2.2 MB [file spectrum.00735-22-s0001.pdf]

Host-age prediction from fecal microbiota composition in male C57BL/6J mice

Adrian Low, Melissa Soh, Sou Miyake, Henning Seedorf

Supplementary Material

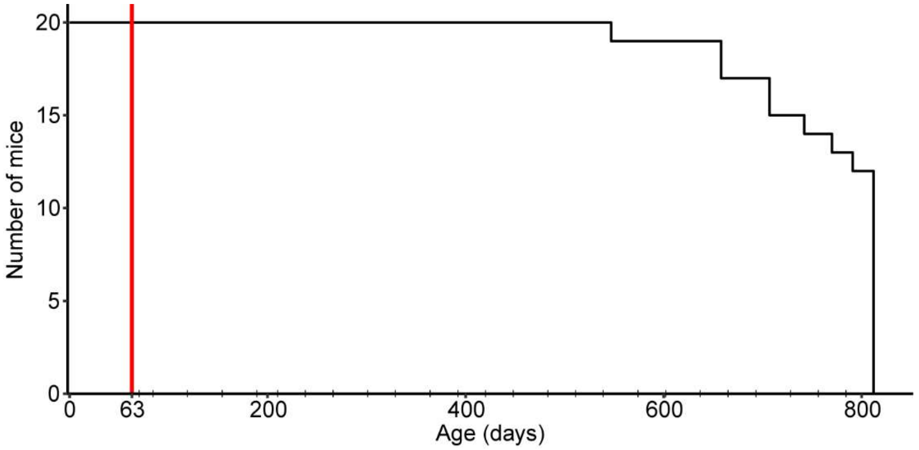

FIG S1 Survival plot of male C57BL/6J mice throughout the study. The red line indicates when the mice arrived at the housing facility and when the first fecal samples were collected. Tick marks on x-axis indicate sampling time points.

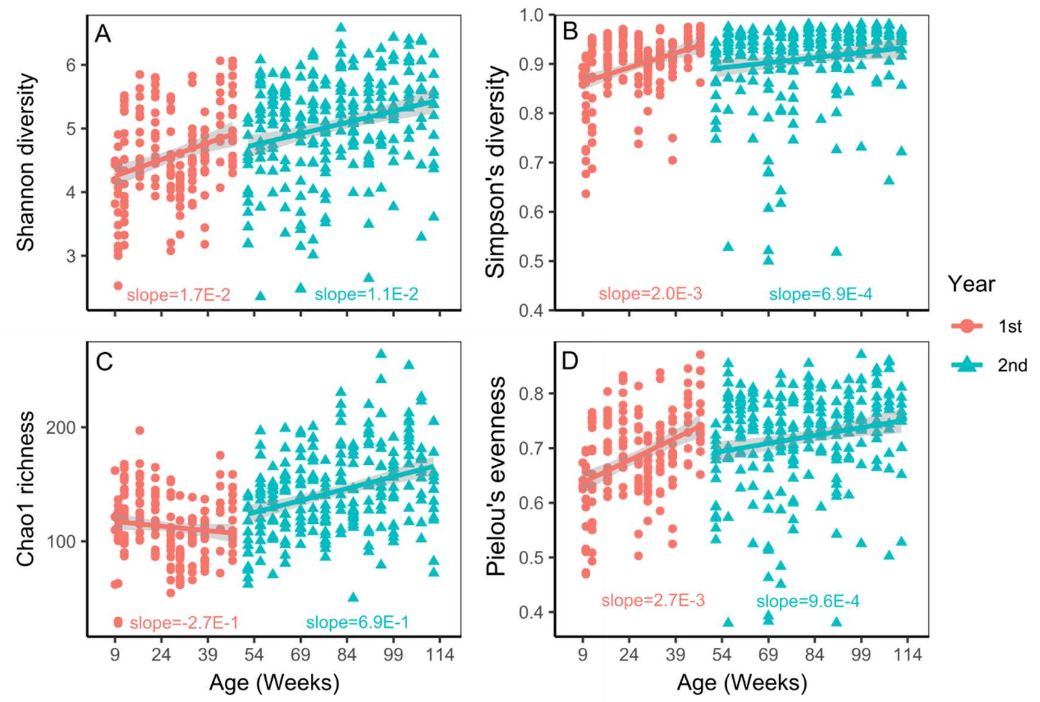

FIG S2. Comparison of alpha-diversity trajectories by year 1 (9-47 weeks) and year 2 (52-112 weeks old). (A) Shannon index, (B) Simpson's diversity, (C) Chao1 richness and (D) Pielou's evenness. Lines are linear mixed-effects regression lines with 95% confidence intervals (grey). Statistics for intercepts and covariance are shown in Table S1F.

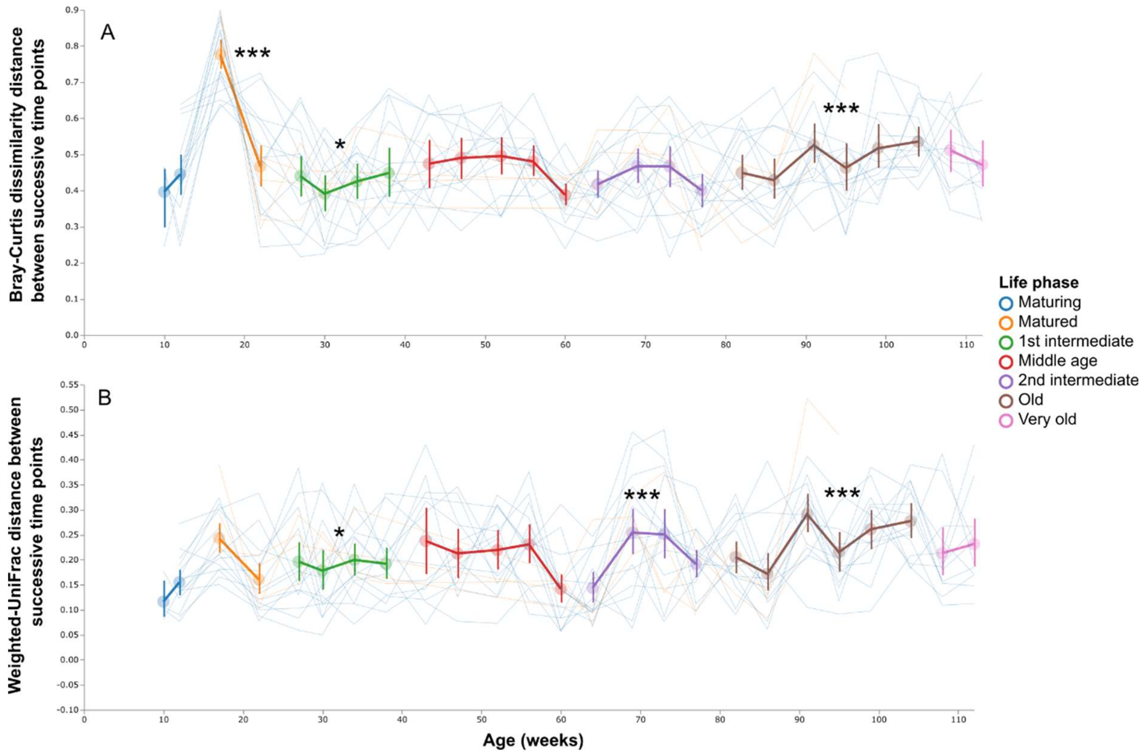

FIG S3. Volatility plots showing the beta-diversity first-distance between successive time points from 9-112 weeks old mice. (A) Bray-Curtis and (B) weighted-UniFrac first-distance. Statistical results for linear mixed-effects models for life phase and time as fixed effects are shown in Table S2A. Significance is denoted with asterisks where (*) denotes *P*-values < 0.05 and (***) denotes *P*-values < 0.001.

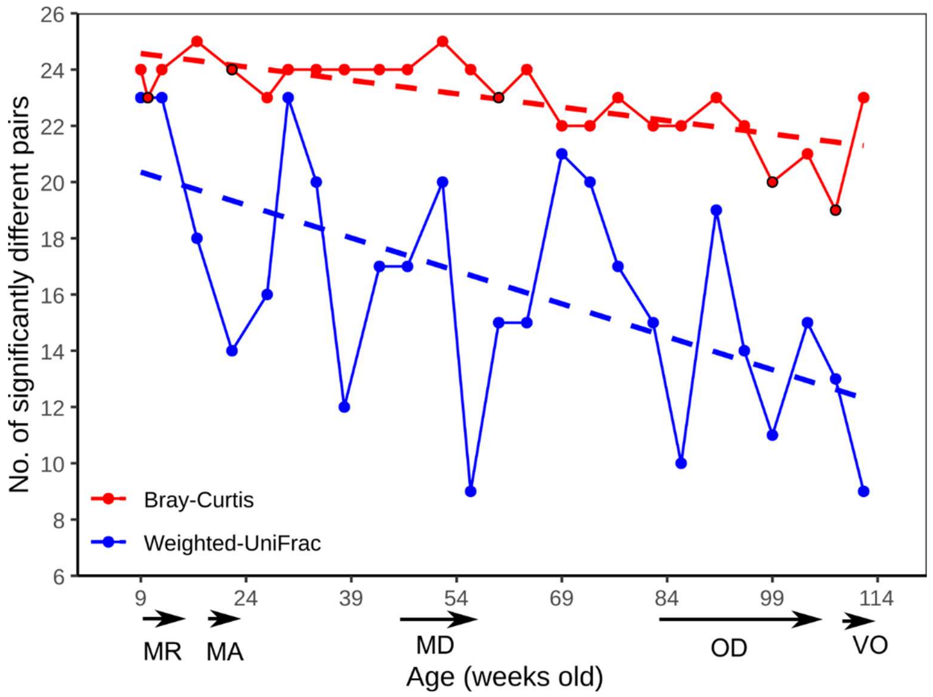

FIG S4. Frequency of significantly different pairs across the fecal microbiota from 9 to 112 weeks old mice based on PERMANOVA analysis of Bray-Curtis dissimilarity and weighted-UniFrac distance matrices. Black outline around Bray-Curtis points are time points used as a training set for SourceTracker.'Maturing' (weeks 9-12; *n* =4-18), 'mature' (weeks 17-22; *n* =19), 'middle age' (weeks 43-60; *n*=11-19), 'old' (weeks 82-104; *n*=15-18) and 'very old' (weeks 108-112; *n*=12-13). 'MR' denotes maturing, 'MA' denotes mature, 'MD' denotes middle age, 'OD' denotes old, and 'VO' denotes very old. Dotted lines indicate the line of best fit for each group.

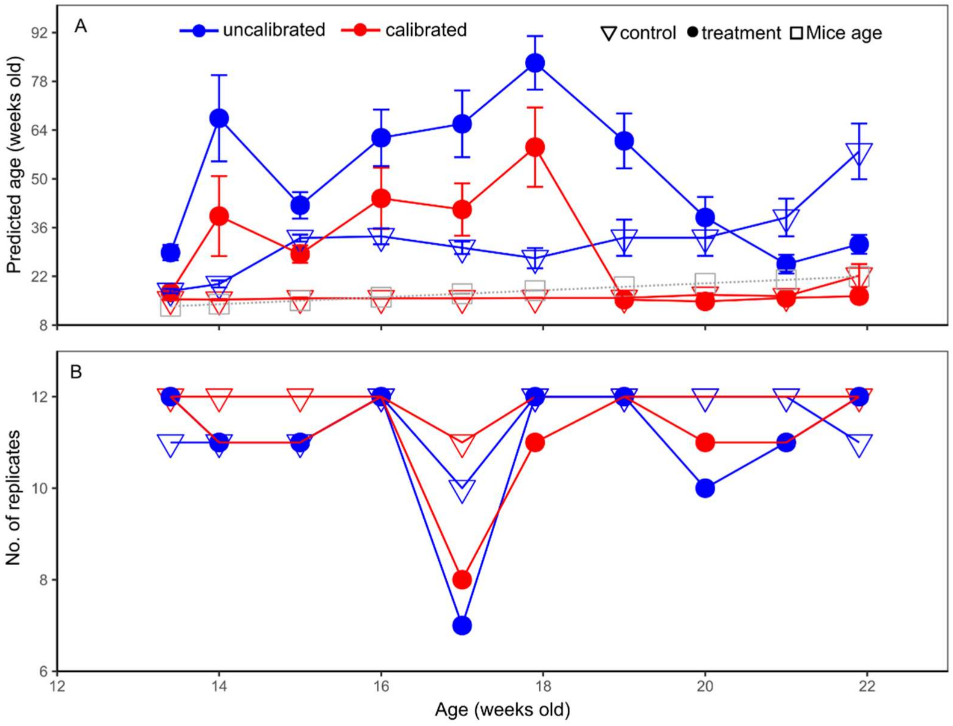

FIG S5. Host-age prediction of mice of dietary treatment study comparing calibrated and uncalibrated training sets. (A) Age prediction of control and treatment mice using uncalibrated and calibrated training sets. The average predicted age is shown by the symbols and error bars are standard error of the mean. (B) Number of replicates after 30% unknown cutoff applied to sinks.

Table S1A Wilcoxon signed-rank test of Shannon diversity index between life phases.

	FDR-corrected P-value
Maturing × Mature (n=18)	0.228752
Mature × Middle Age (n=19)	0.064682
Middle Age × Old (n=19)	0.275341
Old × Very Old (n=13)	1

Fonts in red indicate significant difference (FDR-corrected *P* < 0.05).

Table S1B Wilcoxon signed-rank test of Simpson's diversity index between life phases.

	FDR-corrected P-value
Maturing × Mature (n=18)	2.58E-03
Mature × Middle Age (n=19)	0.322266
Middle Age × Old (n=19)	0.984322
Old × Very Old (n=13)	0.735352

Fonts in red indicate significant difference (FDR-corrected *P* < 0.05).

Table S1C Wilcoxon signed-rank test of Chao1 index between life phases.

Chao1 richness	FDR-corrected P-value
Maturing × Mature (n=18)	0.639694
Mature × Middle Age (n=19)	0.56776
Middle Age × Old (n=19)	1.89E-02
Old × Very Old (n=13)	0.160645

Fonts in red indicate significant difference (FDR-corrected *P* < 0.05).

Table S1E Wilcoxon signed-rank test of Pielou's evenness index between life phases.

Pielou's evenness	FDR-corrected P-value
Maturing × Mature (n=18)	1.12E-02
Mature × Middle Age (n=19)	0.352459
Middle Age × Old (n=19)	0.798252
Old × Very Old (n=13)	0.820312

Fonts in red indicate significant difference (FDR-corrected *P* < 0.05).

Table S1F. Linear mixed-effects model results for the effects of Year 1 (9-47 weeks old mice) vs Year 2 (50-112 weeks old mice) on alpha-diversity.

Model	Variables/Parameters	Estimates (coefficient)	Standard Error	z	*P*-Value
Fixed effects (Shannon ~ Age * Year)	Intercept	4.108	0.158	26.017	0.000
	Year[T.2nd]	0.003	0.249	0.011	0.991
	Age	0.017	0.006	2.74	0.006
	Age:Year[T.2nd]	-0.006	0.005	-1.072	0.284
	Group variance	0.474			
Random effects	Intercept (mouse)	0			
	Slope (Age)	0			
	Covariance (intercept, Age)	0			
Fixed effects (Simpson ~ Age * Year)	Intercept	0.842	0.017	49.705	0.000
	Year[T.2nd]	0.011	0.025	0.446	0.656
	Age	0.002	0.001	3.042	0.002
	Age:Year[T.2nd]	-0.001	0.001	-2.474	0.013
	Group variance	0.005			
Random effects	Intercept (mouse)	0			
	Slope (Age)	0			
	Covariance (intercept, Age)	0			
Fixed effects (Chao1 ~ Age * Year)	Intercept	120.768	7.589	15.914	0.000
	Year[T.2nd]	-32.228	10.903	-2.956	0.003
	Age	-0.259	0.269	-0.965	0.335
	Age:Year[T.2nd]	0.96	0.23	4.176	0.000
	Group variance	907.695			
Random effects	Intercept (mouse)	18.382			
	Slope (Age)	0.616			
	Covariance (intercept, Age)	-0.034			
Fixed effects (Pielou ~ Age * Year)	Intercept	0.612	0.017	35.08	0.000
	Year[T.2nd]	0.03	0.028	1.063	0.288
	Age	0.003	0.001	3.743	0.000
	Age:Year[T.2nd]	-0.002	0.001	-3.012	0.003
	Group variance	0.006			
Random effects	Intercept (mouse)	0			
	Covariance (intercept, Age)	0			
	Slope (Age)	0			

Fonts In red indicate significant covariates (*P* < 0.05)

Table S2A. Linear mixed-effects model results for the effects of beta-diversity difference in distance between successive time points from 9 to 112 weeks old mice.

Model	Variables/Parameters	Estimates	Standard E	z	*P*-Value
Fixed effects (Difference in Bray-Curtis dissimilarity distance ~ Age * Life phase)	Intercept	0.716	0.116	6.177	0.000
	Life phase[T.intermediate1]	-0.368	0.153	-2.401	0.016
	Life phase[T.intermediate2]	-0.229	0.215	-1.063	0.288
	Life phase[T.mature]	1.101	0.181	6.093	0.000
	Life phase[T.maturing]	-0.494	0.378	-1.308	0.191
	Life phase[T.old]	-0.649	0.178	-3.645	0.000
	Life phase[T.very_old]	0.924	1.192	0.776	0.438
	Age	-0.005	0.002	-2.363	0.018
	Age:Life phase[T.intermediate1]	0.007	0.004	1.853	0.064
	Age:Life phase[T.intermediate2]	0.004	0.003	1.265	0.206
	Age:Life phase[T.mature]	-0.057	0.007	-7.693	0.000
	Age:Life phase[T.maturing]	0.022	0.031	0.716	0.474
	Age:Life phase[T.old]	0.01	0.003	3.646	0.000
	Age:Life phase[T.very_old]	-0.005	0.011	-0.494	0.621
	Group variance	0.011			
Random effects	Intercept (mouse)	0			
	Slope (Age)	0			
	Covariance (intercept, Age)	0			
Fixed effects (Difference in Bray-Curtis dissimilarity distance ~ Age * Life phase)	Intercept	0.463	0.088	5.256	0.000
	Life phase[T.intermediate1]	-0.289	0.117	-2.469	0.014
	Life phase[T.intermediate2]	-0.516	0.164	-3.145	0.002
	Life phase[T.mature]	0.05	0.138	0.365	0.715
	Life phase[T.maturing]	-0.48	0.289	-1.661	0.097
	Life phase[T.old]	-0.586	0.136	-4.316	0.000
	Life phase[T.very_old]	-0.744	0.909	-0.818	0.413
	Age	-0.005	0.002	-3.017	0.003
	Age:Life phase[T.intermediate1]	0.005	0.003	1.831	0.067
	Age:Life phase[T.intermediate2]	0.009	0.003	3.383	0.001
	Age:Life phase[T.mature]	-0.011	0.006	-2.002	0.045
	Age:Life phase[T.maturing]	0.019	0.024	0.795	0.427
	Age:Life phase[T.old]	0.009	0.002	4.423	0.000
	Age:Life phase[T.very_old]	0.01	0.008	1.135	0.257
	Group Var	0.006			
Random effects	Intercept (mouse)	0			
	Slope (Age)	0			
	Covariance (intercept, Age)	0			

Font In red indicate significant covariates ($P < 0.05$)

Table S2B PERMANOVA test using Bray-Curtis dissimilarity between life phases.

Group 1	Group 2	Sample si	Permutatic	pseudo-F	p-value	FDR P-value
maturing	mature	75	9999	58.54	0.0001	0.0001
	middle age	118	9999	127.18	0.0001	0.0001
	old	139	9999	124.61	0.0001	0.0001
	very old	62	9999	75.18	0.0001	0.0001
mature	middle age	119	9999	16.72	0.0001	0.0001
	old	140	9999	24.71	0.0001	0.0001
	very old	63	9999	13.44	0.0001	0.0001
middle age	old	183	9999	20.94	0.0001	0.0001
	very old	106	9999	9.28	0.0001	0.0001
old	very old	127	9999	2.14	0.0127	0.0127

Fonts in red indicate significant difference (FDR-corrected $P < 0.01$).

Table S2C PERMANOVA test using weighted-UniFrac distance between life phases.

Group 1	Group 2	Sample si	Permutatic	pseudo-F	p-value	FDR P-value
	mature	75	9999	28.81	0.0001	0.0002
	middle age	118	9999	42.65	0.0001	0.0002
	old	139	9999	44.4	0.0001	0.0002
maturing	very old	62	9999	31.61	0.0001	0.0002
	middle age	119	9999	5.91	0.0008	0.0010
	old	140	9999	10.98	0.0001	0.0002
mature	very old	63	9999	5.65	0.0003	0.0004
	old	183	9999	13.3	0.0001	0.0002
middle age	very old	106	9999	5.15	0.0024	0.0027
old	very old	127	9999	0.84	0.46	0.46

Fonts in red indicate significant difference (FDR-corrected $P < 0.01$).

Table S2D PERMANOVA test using Bray-Curtis dissimilarity between timepoints.

	maturing			mature						middle age									old						very old	
	9	10	12	17	22	27	30	34	38	43	47	52	56	60	64	69	73	77	82	86	91	95	99	104	108	112
9																										
10	0.037																									
12	0.005	0.0144																								
17	0.000	0.0001	0.0001																							
22	0.000	0.0001	0.0001	0.0036																						
27	0.000	0.0001	0.0001	0.0006	0.0439																					
30	0.000	0.0001	0.0001	0.0001	0.0006	0.0451																				
34	0.000	0.0001	0.0001	0.0001	0.0007	0.0051	0.0089																			
38	0.000	0.0001	0.0001	0.0001	0.0001	0.0004	0.0011	0.0803																		
43	0.001	0.0001	0.0001	0.0004	0.0003	0.0001	0.0001	0.0001	0.0010																	
47	0.000	0.0001	0.0001	0.0001	0.0001	0.0001	0.0001	0.0001	0.0001	0.0396																
52	0.001	0.0001	0.0001	0.0001	0.0001	0.0001	0.0003	0.0001	0.0032	0.0001	0.0001															
56	0.000	0.0001	0.0001	0.0001	0.0001	0.0001	0.0001	0.0001	0.0001	0.0003	0.0004	0.0006														
60	0.000	0.0001	0.0001	0.0001	0.0001	0.0001	0.0001	0.0001	0.0001	0.0001	0.0004	0.0001	0.0887													
64	0.000	0.0001	0.0001	0.0001	0.0001	0.0001	0.0001	0.0001	0.0001	0.0001	0.0001	0.0001	0.0076	0.0718												
69	0.000	0.0001	0.0001	0.0001	0.0001	0.0001	0.0001	0.0001	0.0001	0.0001	0.0001	0.0003	0.0004	0.0003	0.0011											
73	0.000	0.0001	0.0001	0.0001	0.0001	0.0001	0.0001	0.0001	0.0001	0.0001	0.0001	0.0001	0.0001	0.0001	0.0001	0.1058										
77	0.000	0.0001	0.0001	0.0001	0.0001	0.0001	0.0001	0.0001	0.0001	0.0001	0.0001	0.0001	0.0003	0.0001	0.0029	0.0271	0.0297									
82	0.000	0.0001	0.0001	0.0001	0.0001	0.0001	0.0001	0.0001	0.0001	0.0001	0.0001	0.0001	0.0001	0.0001	0.0005	0.0003	0.0001	0.001								
86	0.000	0.0001	0.0001	0.0001	0.0001	0.0001	0.0001	0.0001	0.0001	0.0001	0.0001	0.0001	0.0001	0.0001	0.0007	0.0003	0.0003	0.003	0.0623							
91	0.000	0.0001	0.0001	0.0001	0.0001	0.0001	0.0001	0.0001	0.0001	0.0001	0.0001	0.0001	0.0001	0.0001	0.0001	0.0457	0.0189	0.002	0.0001	0.0003						
95	0.000	0.0001	0.0001	0.0001	0.0001	0.0001	0.0001	0.0001	0.0001	0.0001	0.0001	0.0001	0.0001	0.0001	0.0001	0.0003	0.0004	0.000	0.0035	0.0069	0.0090					
99	0.001	0.0001	0.0001	0.0001	0.0001	0.0001	0.0001	0.0001	0.0001	0.0001	0.0001	0.0001	0.0001	0.0001	0.0001	0.0004	0.0003	0.001	0.0054	0.0887	0.0004	0.06				
104	0.000	0.0001	0.0001	0.0001	0.0001	0.0001	0.0001	0.0001	0.0001	0.0001	0.0001	0.0001	0.0001	0.0001	0.0003	0.0004	0.0013	0.002	0.0221	0.0059	0.0021	0.04	0.03			
108	0.001	0.0001	0.0001	0.0001	0.0001	0.0001	0.0001	0.0001	0.0001	0.0001	0.0001	0.0001	0.0003	0.0001	0.0001	0.0030	0.0055	0.003	0.0341	0.0140	0.0057	0.06	0.04	0.599		
112	0.001	0.0001	0.0001	0.0001	0.0001	0.0001	0.0001	0.0001	0.0001	0.0001	0.0001	0.0001	0.0001	0.0001	0.0001	0.0019	0.0003	0.002	0.0003	0.0057	0.0006	0.00	0.07	0.005	0.04	

Boxes in red indicate significant difference (FDR-corrected $P < 0.01$) between timepoints.

Table S2E PERMANOVA test using weighted-UniFrac distance between timepoints

	maturing			mature						middle age									old						very old	
	9	10	12	17	22	27	30	34	38	43	47	52	56	60	64	69	73	77	82	86	91	95	99	104	108	112
9																										
10	0.035																									
12	0.134	0.0154																								
17	0.001	0.0003	0.0003																							
22	0.002	0.0003	0.0003	0.051																						
27	0.000	0.0003	0.0003	0.038	0.061																					
30	0.001	0.0003	0.0003	0.000	0.000	0.07387																				
34	0.001	0.0003	0.0003	0.005	0.018	0.16583	0.0052																			
38	0.004	0.0003	0.0003	0.010	0.033	0.25656	0.0467	0.484																		
43	0.002	0.0003	0.0003	0.001	0.074	0.00175	0.0003	0.001	0.015																	
47	0.001	0.0003	0.0003	0.001	0.085	0.01191	0.0007	0.004	0.033	0.226																
52	0.000	0.0003	0.0003	0.001	0.001	0.047	0.0072	0.037	0.089	0.001	0.000															
56	0.005	0.0003	0.0003	0.013	0.041	0.01409	0.0003	0.011	0.047	0.072	0.031	0.0065														
60	0.000	0.0003	0.0003	0.001	0.061	0.00241	0.0003	0.002	0.014	0.080	0.075	0.0007	0.321													
64	0.001	0.0003	0.0003	0.001	0.009	0.00271	0.0003	0.003	0.007	0.004	0.002	0.0026	0.279	0.254												
69	0.003	0.0003	0.0003	0.000	0.000	0.00033	0.0003	0.000	0.002	0.001	0.000	0.0080	0.003	0.000	0.001											
73	0.000	0.0003	0.0003	0.000	0.000	0.00033	0.0003	0.000	0.000	0.000	0.000	0.0005	0.004	0.000	0.000	0.203										
77	0.001	0.0003	0.0003	0.001	0.001	0.0016	0.0003	0.001	0.004	0.000	0.000	0.0179	0.009	0.001	0.033	0.030	0.007									
82	0.001	0.0003	0.0003	0.002	0.005	0.00033	0.0003	0.000	0.000	0.026	0.002	0.0005	0.229	0.052	0.061	0.001	0.001	0.002								
86	0.003	0.0003	0.0003	0.012	0.043	0.00462	0.0003	0.003	0.027	0.044	0.015	0.0007	0.229	0.267	0.229	0.001	0.001	0.013	0.147							
91	0.001	0.0003	0.0003	0.001	0.000	0.00033	0.0003	0.000	0.001	0.000	0.000	0.0013	0.003	0.000	0.001	0.476	0.432	0.013	0.001	0.001						
95	0.002	0.0003	0.0003	0.002	0.001	0.00033	0.0003	0.001	0.001	0.003	0.000	0.0007	0.119	0.006	0.038	0.009	0.040	0.015	0.245	0.088	0.038					
99	0.001	0.0003	0.0003	0.011	0.047	0.00393	0.0003	0.002	0.023	0.060	0.016	0.0013	0.150	0.110	0.047	0.001	0.001	0.005	0.110	0.597	0.001	0.061				
104	0.001	0.0003	0.0003	0.003	0.001	0.00033	0.0003	0.001	0.001	0.002	0.001	0.0005	0.106	0.007	0.041	0.006	0.033	0.008	0.417	0.105	0.013	0.583	0.080			
108	0.003	0.0003	0.0003	0.001	0.001	0.00033	0.0003	0.000	0.001	0.003	0.001	0.0005	0.141	0.005	0.035	0.020	0.078	0.010	0.336	0.066	0.038	0.719	0.055	0.835		
112	0.001	0.0003	0.0003	0.038	0.022	0.07572	0.0021	0.042	0.110	0.001	0.004	0.0398	0.083	0.022	0.071	0.007	0.001	0.075	0.011	0.275	0.002	0.021	0.239	0.015	0.016	

Boxes in red indicate significant difference (FDR-corrected $P < 0.01$) between timepoints.

Table S4 Wilcoxon signed-rank test of relative abundance between Firmicutes and Bacteroidota for each time point.

Bacteroidota/Firmicutes	FDR-corrected P-value
9-week-old	0.12000
10-week-old	0.07300
12-week-old	0.90000
17-week-old	0.40000
22-week-old	0.01400
27-week-old	0.00200
30-week-old	0.00002
34-week-old	0.01200
38-week-old	0.04400
43-week-old	0.05400
47-week-old	0.00043
52-week-old	0.15000
56-week-old	0.70000
60-week-old	0.00820
64-week-old	0.12000
69-week-old	0.00830
73-week-old	0.00042
77-week-old	0.63000
82-week-old	0.55000
86-week-old	0.14000
91-week-old	0.00160
95-week-old	0.04400
99-week-old	0.25000
104-week-old	0.21000
108-week-old	0.08000
112-week-old	0.06400

Fonts in red indicate significant difference (FDR-corrected $P < 0.05$).
